# Sequencing of cerebrospinal fluid in non‐small‐cell lung cancer patients with leptomeningeal metastasis: A systematic review

**DOI:** 10.1002/cam4.5163

**Published:** 2022-08-24

**Authors:** Tianqi Gao, Fengxi Chen, Man Li

**Affiliations:** ^1^ Department of Oncology The Second Hospital of Dalian Medical University Dalian China

**Keywords:** gene copy number, metastasis, next‐generation sequencing, non‐small‐cell lung cancer

## Abstract

Leptomeningeal metastasis (LM) refers to the dissemination of malignant cells in the subarachnoid space, pia, and arachnoid mater and is a severe condition associated with metastatic solid tumors. The most common solid tumor that develops into LM is lung cancer and the incidence increased in patients with advanced non‐small‐cell lung cancer (NSCLC) with targetable mutations. However, tissue biopsy of LM is inaccessible, leading to the paucity of genomic profiles of LM to guide targeted treatments and explore biological mechanisms. In recent years, liquid biopsy is considered a minimally invasive and dynamic method to trace the genomic alterations of cancer cells and some studies started to perform sequencing of cerebrospinal fluid (CSF) in patients with LM to reveal the targeted mutations and genomic profiles. In this review, we focused on studies performed sequencing of CSF in NSCLC patients with LM and summarized the sequencing results and their commonality. As the only way to reveal the genomic landscapes of LM, our review provided evidence that sequencing of CSF is a promising management method in LM patients to dynamically guide target therapy and monitor intracranial tumor response. Furthermore, it reveals a unique genomic profile of LM including driver genes, drug‐resistant mutations, and a number of copy number variations. Sequencing of CSF in LM patients seems to provide more comprehensive genomic information than we expected and the biological significance behind the genomic alternations needs further study.

## Graphical abstract

The frequency of CNVs and alternations of the reported genes.

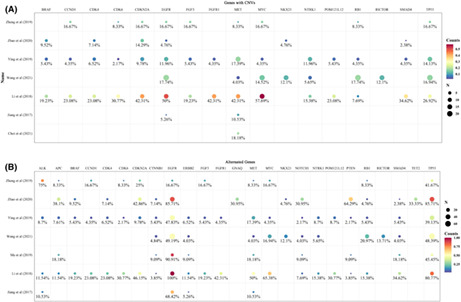

## 1 INTRODUCTION

Leptomeningeal metastasis (LM) is a severe condition associated with metastatic solid tumors and refers to the dissemination of malignant cells in the subarachnoid space, pia, and arachnoid mater.^1^ The most common solid tumors that develop into LM are lung cancer, breast cancer, and melanoma. The incidence of LM in patients with advanced non‐small‐cell lung cancer (NSCLC) was 3%–5% and increased in subgroups of patients with targetable mutations due to the prolonged survival through better‐tolerated and more effective therapies.^2^ However, detecting targeted alternations of LM is challenged because the genomic landscape changes from primary extracranial lesions. Yet, tissue biopsy of LM is inaccessible due to its diffused distribution and the risk of potential complications caused by intracranial surgical procedures. The paucity of genomic profiles leads to a disadvantaged treatment condition for LM patients and a poor understanding of the potential biological mechanisms of LM.

Liquid biopsy, which refers to the isolation and analysis of tumor‐derived materials from blood or other bodily fluids, is a minimally invasive and dynamic method to trace the genomic alterations of cancer cells.^3^, ^4^ Previous studies have indicated that cerebrospinal fluid (CSF) is a more reliable material than plasma in central nervous system (CNS) tumors, with a higher detection rate of mutations and less interference from normal blood cells.^5^, ^6^, ^7^, ^8^, ^9^ Some studies also performed sequencing of CSF to investigate the genomic alterations in NSCLC patients with LM, but with a limited number of patients. Thus, limited information is available on the genomic landscape and the application of CSF sequencing in LM patients. Here, we present a systematic review of the studies on sequencing of CSF in NSCLC patients with LM, and the objectives were to (1) evaluate the performance of CSF as a sequencing medium for LM patients, (2) describe the characteristics of the genomic profile of LM, (3) summarize its clinical application in guiding target treatments, and (4) to discuss the clinical relevance and the potential biological significance of the genomic alterations.

## 2 METHODS

### 2.1 Data collection

This study was conducted according to the Preferred Reporting Items for Systematic Reviews and Meta‐analysis (PRISMA) guidelines.^10^ Because there were variations in the patients, sequencing methods, and reported genes in each study, a full meta‐analysis was not feasible and a descriptive synthesis was performed. Studies were identified by searching the PubMed and Medline electronic databases from the date of inception to December 31, 2021. Conference articles were also searched on the websites of the American Society of Clinical Oncology and the European Society for Medical Oncology. Search strategies are presented in the Supplementary File. The inclusion criteria were (i) original studies, (ii) studies comprised patients diagnosed with LM, (iii) type of the primary tumor was NSCLC or NSCLC was included, (iv) sequencing samples were from CSF, (v) the study included more than one patient who meet criteria (ii), (iii), and (iv). The exclusion criteria were (i) reviews, meta‐analyses, comment articles or case reports, (ii) no patient with NSCLC, (iii) non‐English articles, (iv) incomplete information, and (v) duplicate publication. Two authors independently evaluated the eligibility of the studies. Study quality was appraised by using the Joanna Briggs Institute Prevalence Critical Appraisal Tool.^11^

### 2.2 Data extraction

Data were extracted from all the available text, figures, tables, and supplementary documents from the studies and compiled into predesigned extraction forms. Information regarding the following characteristics was extracted: author, year, institution, topic, number of patients, treatment history of the tyrosine kinase inhibitors (TKI), sequencing platform and method, sequencing sample, paired plasma, and paired tissue. Alternated genes were analyzed per study and on a per patient basis. Alternations, including single nucleotide variations (SNVs), insertion–deletion mutations, frameshift mutations, and copy number variations (CNVs) of the reported genes, were obtained. When the mutations were not included in the above types, the data were excluded. The results of multiple sampling at the same time and different sequencing samples from one patient were considered as a whole, which were analyzed together to represent the genomic alterations of the patient. The detection rates and the concordance rates were recorded if they were reported in the original studies. If the rates were not reported explicitly, they were presented in the form of a ratio through manual extraction. We compared the alternated genes with the list of Cancer Gene Census (CGC) and pan‐lung cancer data in The Cancer Genome Atlas (TCGA) database. CGC is an ongoing effort to catalog alternated genes that have been causally implicated in cancer and explain how the dysfunction of these genes drives cancer. The data can be obtained from the websites directly. Representative cases of CSF sequencing in guiding treatment were also summarized.

### 2.3 Statistical analysis

Only descriptive statistics were used. The frequency was defined as the number of patients with detected mutations divided by the total number of patients.

## 3 RESULTS

### 3.1 Study details

**FIGURE 1:**
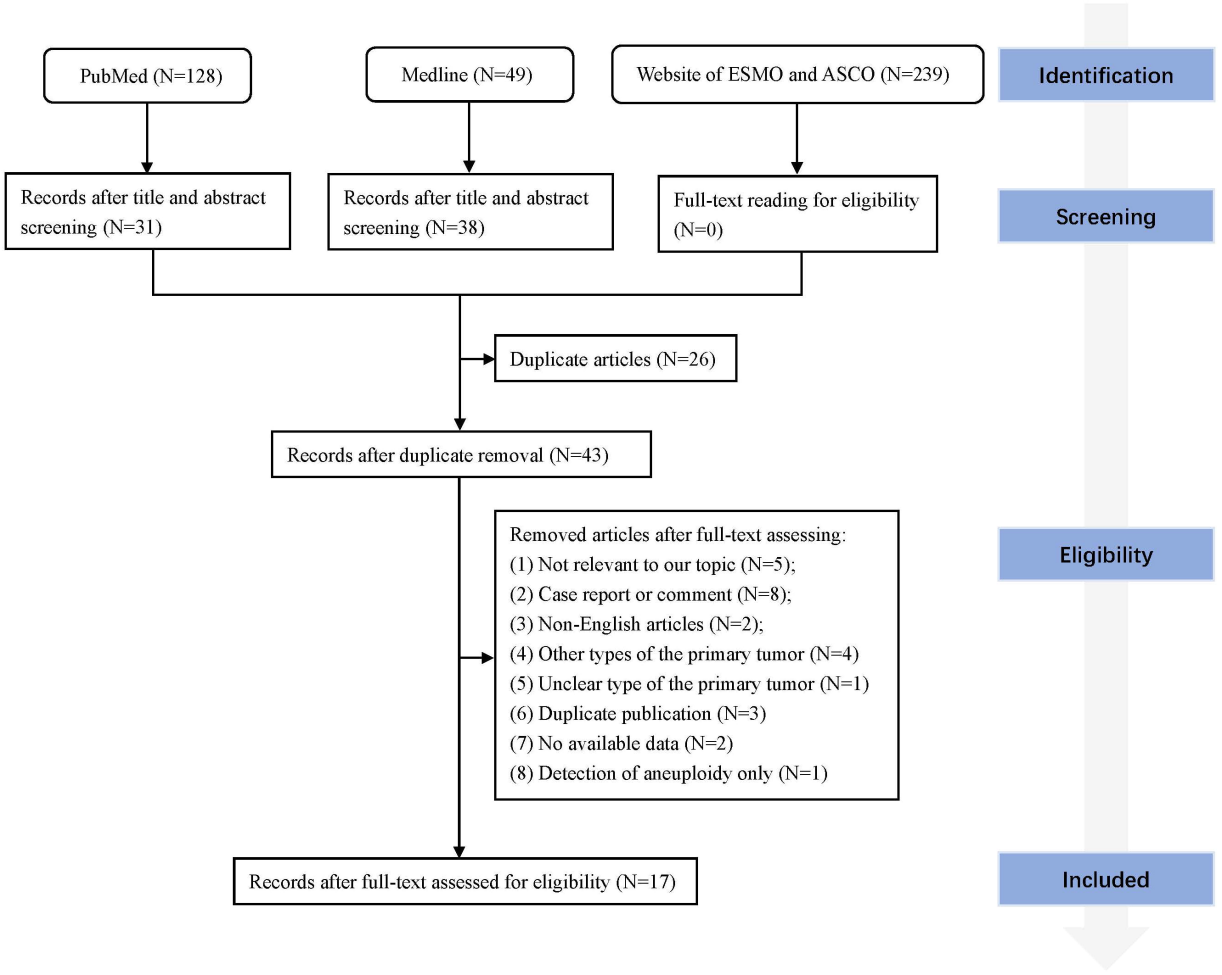
Flowchart of selection of the included studies.

A total of 17 articles were included in this review according to the eligibility criteria through full‐text reading^8^, ^9^, ^12^, ^13^, ^14^, ^15^, ^16^, ^17^, ^18^, ^19^, ^20^, ^21^, ^22^, ^23^, ^24^, ^25^, ^26^ (Figure 1). Eleven patients in two studies reported by Ma et al. were duplicates but the study content was different.^13^, ^22^ One study^22^ only described the subtypes of epidermal growth factor receptor (*EGFR*) mutations and the other^13^ reported all the mutated genes but without illustrations in *EGFR* subtypes, so the information of the two studies was merged and duplicated patients were removed. Patients with other types of primary tumors were also reported in three studies^15^, ^17^, ^26^ and those patients were excluded. In total, sequencing results from 541 patients in 17 studies were analyzed. Five studies specifically included patients with *EGFR* mutations in the primary tumor and one study included patients with ALK receptor tyrosine kinase (*ALK*) fusions. The history of TKI treatments was illustrated in 238 patients and 192 of them were TKI exposed. Three hundred and twenty‐two patients had sequencing results from paired plasma, and 212 patients had sequencing results from paired biopsy tissues. Eight studies performed sequencing through different cancer‐related gene panels, and nine studies sequenced only predesignated genes including *EGFR*, KRAS proto‐oncogene (*KRAS*), B‐Raf proto‐oncogene (*BRAF*), erb‐b2 receptor tyrosine kinase 2 (*ERBB2*), and MET proto‐oncogene (*MET*). Cell‐free DNA (cfDNA) in CSF was sequenced in 14 studies, cells in CSF were sequenced in four studies, and circulating tumor cells (CTC) in CSF were sequenced in three studies. The next‐generation sequencing (NGS) and the droplet digital PCR (ddPCR) were the most commonly used methods and were used in 10 and five studies, respectively. Most studies used various Illumina platforms for NGS and BioRad QX20TM system for ddPCR (Table 1).

**TABLE 1:** Characteristics of the included studies

Study (year)	Topic	Institution	Study design	Patients	Patient inclusion criteria	Sample type	Target genes	Method	Platform	TKI‐naive	TKI exposed	Paired plasma	Paired tissue
Jiang et al. (2017)	To determine the effectiveness of the CellSearch assay and identify the gene profiles of CSFCTCs in LM patients	Guangdong Lung Cancer Institute; Guangdong General Hospital	Prospective	19	NA	CSFCTC	416 cancer‐related gene panel	NGS	Illumina HiSeq 4000	3	16	0	11
Li et al. (2018)	To provide evidence of the clinical utility of CSF as a liquid biopsy medium in LM of *EGFR*‐mutant NSCLC	Guangdong Lung Cancer Institute	Prospective	28	*EGFR*‐mutated patients	CSFcfDNA; CSF precipitates	168 lung cancer‐related genes	NGS	Illumina Nextseq 500	3	25	26	13
Ma et al. (2019)	To compare the results from CSF ctDNA with plasma ctDNA and plasma circulating tumor cells	Tianjin Huanhu Hospital	Prospective	11	NA	CSFCTC	Ion AmpliSeq Cancer HotSpot Panel v2	NGS	Ion Proton system	1	10	11	0
Zheng et al. (2019)	To reveal clinically targetable genomic alterations and resistance mechanisms of LM and uncover patterns of tumor response in *ALK*‐rearranged NSCLC	Guangdong Lung Cancer Institute	Prospective	11	*ALK*‐rearranged patients	CSFcfDNA; CSF precipitates	168 lung cancer‐related genes	NGS	Illumina HiSeq 5000	3	8	11	3
Ying et al. (2019)	To evaluate the ability of both liquid biopsy media; CSF and plasma in repopulating the genomic profile of LM	Taizhou First People's Hospital; Taizhou University Hospital; Nantong University Affiliated Hospital or Second Affiliated Hospital of Fujian Medical University	Prospective	92	NA	CSFcfDNA	168 lung cancer‐related genes	NGS	Illumina HiSeq 5000	NK	NK	72	0
Zhao et al. (2020)	To characterize neoplastic meningitis genetic profiles and profile of gene mutations	The Second Hospital of Hebei Medical University	Prospective	42	NA	CSFcfDNA	143 cancer‐related genes	NGS	Ion Proton system	NK	NK	0	0
Wang et al. (2021)	To reveal the genetic alterations of CSF and compare the genetic difference of CSF with matched plasma and tissue samples	Nanjing Drum Tower Hospital and other medical centers were enrolled	Prospective	124	NA	CSFcfDNA	416 cancer‐related genes	NGS	Illumina HiSeq 4000	NK	NK	72	24
Frankel et al. (2018)	To investigate the feasibility and clinical relevance of *EGFR*, *KRAS*, *BRAF*, and *HER2* molecular testing from CSF malignant cells	Assistance Publique des Hôpitaux de Marseille	Retrospective	5	NA	CSF cells	*EGFR*, *KRAS*, *BRAF*, and *ERBB2*	qPCR, Sanger	Roche LightCycler 480, Applied Biosystems 3500 or 3130 Dx Genetic Analyzer	NK	NK	1	3
Xu et al. (2018)	To exploit CSF samples to systematically investigate the molecular profiles of LM secondary to NSCLC	Peking Union Medical College Hospital	Prospective	49	NA	CSFcfDNA	*EGFR*	ARMS, ddPCR	NK	16	26	0	0
Ge et al. (2019)	To explore whether the tumor‐associated mutations can be detected by different next‐generation sequencing pipelines in paired CSF and plasma samples from lung adenocarcinoma patients with LM	Huashan Hospital Fudan University	Prospective	18	NA	CSF cfDNA, CSF cells	Different panels	NGS, ddPCR	Illumina NextSeq 500, Hiseq 4000, MiSeq, or MiniSeq; QX200TM ddPCR System	NK	NK	18	9
Li et al. (2019)	To assess the diagnosis, treatment modes, and survival status of *EGFR*‐mutant NSCLC patients with LM	Cancer Hospital, Chinese Academy of Medical Sciences, and Peking Union Medical College	Retrospective	32	*EGFR*‐mutated patients	CSFcfDNA	,	ARMS, NGS	NK	5	27	29	29
Huang et al. (2019)	To explore the *EGFR* status in CNS metastases of lung adenocarcinoma patients and to guide the treatment of intracranial and extracranial tumors in these patients	Huashan Hospital, Fudan University	Prospective	15	Patients with known *EGFR* status	CSFcfDNA	*EGFR*	ddPCR	BioRad QX20TM ddPCR System	NK	NK	14	15
Ma et al. (2020)	To evaluate the *EGFR* mutations in patients with NSCLC and newly diagnosed brain metastasis and to examine the effect of *EGFR* tyrosine kinase inhibitors on brain metastasis harboring CSF‐tested uncommon EGFR mutations	Tianjin Huanhu Hospital	Prospective	11	NA	CSFcfDNA	*EGFR*	NGS	Ion Torrent S5XL	1	10	0	10
Chiang et al. (2021)	To evaluate the utility of CSF as a medium for EGFR mutation testing in clinical practice.	NK	Prospective	51	*EGFR*‐mutated patients	CSFcfDNA	*EGFR*	*EGFR* mutation assay kit	Roche cobas *EGFR* Mutation Test v2	3	48	37	51
Bussel et al. (2020)	To determine the sensitivity and specificity of epithelial cell adhesion molecule immune flow cytometry circulating tumor cells analysis in CSF in patients with suspected LM and to explore the distribution of driver mutations in different samples	Netherlands Cancer Institute–Antoni van Leeuwenhoek and the Medical CenterSlotervaart	Prospective	7	NA	CSFcfDNA, CSFCTC	*EGFR*	ddPCR	BioRad QX20TM ddPCR System	NK	NK	5	7
Choi et al. (2020)	To evaluate whether genotyping cfDNA in the CSF may be helpful in managing LM of *EGFR*‐mutant NSCLC	National Cancer Center Hospital	Prospective	11	*EGFR*‐mutated patients	CSFcfDNA	*EGFR*, *MET*	Nanowire assay	—	0	11	11	11
Liu et al. (2021)	To determine the ability of CSF compared to plasma in detecting *EGFR* mutation in patients with lung adenocarcinoma and CNS metastasis	Henan Cancer Hospital	Prospective	26	*EGFR*‐mutated patients	CSFcfDNA	*EGFR*	ddPCR	BioRad QX20TM ddPCR System	5	21	26	26

*Note*: The numbers in the table represent the number of patients. The column of patients represents the included non‐small‐cell lung cancer patients with LM who performed sequencing of CSF.

Abbreviations: ARMS, amplification refractory mutation system; cfDNA, cell‐free DNA; CNS, central nervous system; CSF, cerebrospinal fluid; ctDNA, cell‐free tumor DNA; CTC, circulating tumor cells; ddPCR, droplet digital PCR; LM, leptomeningeal metastasis; NGS, next‐generation sequencing; NK, not known; NSCLC, non‐small‐cell lung cancer; qPCR, quantitative PCR; TKI, tyrosine kinase inhibitors.

Quality assessment of the included studies was performed according to the Joanna Briggs Institute Prevalence Critical Appraisal Tool (Table S1). Three studies were assessed with a low risk of bias and 13 with a moderate risk of bias. Given the variations in the baseline features of patients, sequencing methods, and targeted genes in each study, the quality of evidence of the included studies in the systematic review was determined to be a high risk of bias.

### 3.2 Performance of CSF as a sequence medium for LM patients

Six studies compared CSF with the primary tumor in driver gene mutations and found the results are highly consistent with each other. Five studies focused on *EGFR* mutations and one study focused on *ALK* fusions. The concordance rate of *EGFR* mutations detected in CSF and the primary tumor was 67.6%–100%.^8^, ^9^, ^12^, ^21^, ^23^, ^25^ One study found the concordance rate of *ALK* was 81.8%.^14^ Seven studies did not report the concordance rate directly but it could be summarized from the tables or figures of each article. The number of patients with matched *EGFR* mutations detected in CSF to the primary tumor were 9/11, 9/10, 3/5, 7/9, 21/29, 5/5, and 7/11, respectively.^13^, ^15^, ^17^, ^19^, ^20^, ^24^, ^26^

Plasma is the most common sample for liquid biopsy and also an alternative for LM. To explore whether there was a superiority of CSF, the studies compared CSF with paired plasma in the detection rates and the average maximum allele fractions (AFs) (Table 2). Four studies compared the detection rate of *EGFR* and found that it was higher in CSF. In *EGFR*‐mutated patients, the detection rate of *EGFR* was 67.6%–100% in CSF and 36.4%–73.1% in plasma.^8^, ^21^, ^23^, ^25^ We can also summarize that the number of samples with detected driver gene mutations in CSF was more than that in a paired plasma sample, even though there were no clear detection rates reported (Table 2). The AFs of mutated genes in CSF were significantly higher than in plasma, which was reported in four studies.^8^, ^9^, ^14^, ^19^ When using gene panels, more alternated genes were demonstrated in CSF than in plasma. Four studies reported a relatively higher mutation detection rate in CSF (defined as at least one mutation was detected) and three of them were statistically significant.^9^, ^16^, ^19^, ^20^ Collectively, we could find that CSF was superior to plasma as a sequence medium of LM (Table 2).

**TABLE 2:** Performance of CSF as a sequence medium for LM patients

Author	Driver genes	Concordance rate with primary tumor		Detection rate of driver genes					Average maximum allele fractions			Detection rates of mutated genes		
		CSF		CSF		Plasma		*p*	CSF	Plasma	*p*	CSF	Plasma	*p*
Jiang et al. (2017)	*EGFR; ALK*	17/19	89.50%	17/19	89.50%	NA	NA	NA	NA	NA	NA	NA	NA	NA
Li et al. (2018)	*EGFR*	26/26	100%	26/26	100%	19/26	73.10%	0.01	62.0%	3.5%	—	NA	NA	NA
Ma et al. (2019)	*EGFR*	9/11	—	9/11	—	3/11	—	—	NA	NA	NA	NA	NA	NA
Zheng et al. (2019)	*ALK*	9/11	81.80%	9/11	81.80%	5/11	45.50%	0.183	NA	NA	0.009	NA	NA	NA
Ying et al. (2019)	*EGFR*	NA	NA	NA	NA	NA	NA	NA	43.64%	4.58%	<0.001	81.5%	62.5%	0.008
Zhao et al. (2020)	*EGFR*	9/10	—	9/10	—	NA	NA	NA	NA	NA	NA	NA	NA	NA
Wang et al. (2021)	*EGFR*	NA	NA	NA	NA	NA	NA	NA	NA	NA	NA	72.22%	81.94%	0.2339
Frankel et al. (2018)	*EGFR; KRAS*	3/5	—	3/5	—	NA	NA	NA	NA	NA	NA	NA	NA	NA
Xu et al. (2018)	*EGFR*	NA	NA	NA	NA	NA	NA	NA	NA	NA	NA	NA	NA	NA
Ge et al. (2019)	*EGFR*	7/9	—	7/9	—	4/16	—	—	49.62%	13.19%	—	80%^a^	30.77%	0.0039^a^
												90.91%^b^		0.0013^b^
Ning Li et al. (2019)	*EGFR*	21/29	—	21/29	—	8/29	—	—	NA	NA	NA	24/32	11/29	0.003
Huang et al. (2019)^2^	*EGFR*	9/12	75.00%	9/12	75.00%	4/11	36.40%	0.062	NA	NA	NA	NA	NA	NA
Chiang et al. (2021)	*EGFR*	35/48	72.90%	25/37	67.60%	20/37	54.05%	0.295	NA	NA	NA	NA	NA	NA
Bussel et al. (2020)	*EGFR*	5/5	—	5/5	—	4/5	—	—	NA	NA	NA	NA	NA	NA
Choi et al. (2021)	*EGFR*	7/11	—	7/11	—	4/11	—	—	NA	NA	NA	NA	NA	NA
Liu et al. (2021)	*EGFR*	22/26	84.60%	22/26	84.60%	10/26	38.50%	0.004	33.50%	1.75%	<0.001	NA	NA	NA

*Note*: The concordance rate with primary tumor means the number of patients with the same driver gene mutations as the primary tumor detected in CSF divided by the number of all patients with the paired primary tumor for sequencing. The detection rate of driver genes in CSF and plasma mean the number of patients with detected driver gene mutations divided by the number of all patients who performed sequencing of CSF or plasma. The percentage was recorded as reported in the originally included studies. If the rates were not reported explicitly, they were presented in the form of a ratio through manual extraction. Two rates were reported, respectively, in Ge et al. (2019): A, cfDNA vs plasma; B, CSF cells vs plasma.

Abbreviations: ‘—‘, not reported; CSF, cerebrospinal fluid; EGFR, epidermal growth factor receptor; LM, leptomeningeal metastasis; NA, not available.

### 3.3 Characteristics of the genomic profile of LM

#### 3.3.1 *EGFR* mutations

**FIGURE 2:**
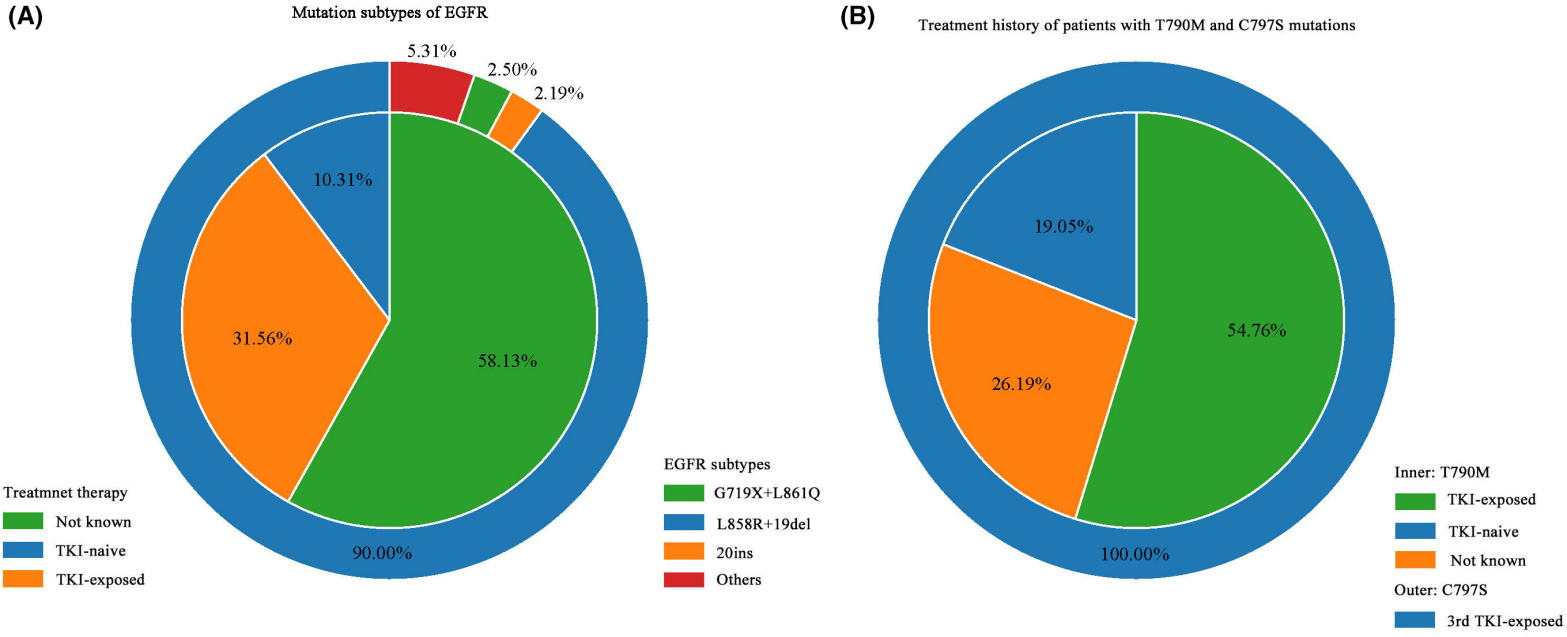
Subtypes of *EGFR* mutations. (A) The outer circle represents subtypes of *EGFR* mutations including L858R, 19del, G719X, L861Q, 20ins, and other types (T790M and C797S were not included). L858R and 19del accounted for 90%. The inner circle represents the treatment history of patients, and 31.56% of patients received TKI treatment. (B) The treatment history of patients with T790M and C797S. The outer circle represents patients with C797S, and all of them received the third‐generation TKI. The inner circle represents patients with T790M, and 54.76% of patients had received TKI treatment. *EGFR*, epidermal growth factor receptor; TKI, tyrosine kinase inhibitors.

A total of 525 patients in 15 studies performed sequencing of CSF and 377 of them had detected *EGFR* mutations. Fourteen studies reported subtypes of *EGFR* mutations. The most frequent subtypes were classical sensitive exon21 L858R and exon19 del mutations, which occurred in 158/377 and 137/351 patients, and accounted for 90% (288/320) of all the activating *EGFR* mutations, which was similar to the prevalence in NSCLC.^27^ The refractory mutation exon20 ins was detected in 7/351 patients. The uncommon mutations G719X and L861Q were detected in 5/360 and 4/360 patients, respectively. E709A, E764V, and G757R were detected in one patient each, while the number was too small to elucidate the relevance to the genomic characteristics of LM (Figure 2A).

The most common secondary mutation T790M was detected in 42/377 patients. Of the 42 patients, the treatment history of 34 patients were known, and 67% (23/34) patients had received TKI treatment. C797S mutations were detected in seven patients and all of them were exposed to the third generation of TKI, indicating the secondary drug‐resistant mutations in CSF were also relevant to TKI treatment (Figure 2B).

#### 3.3.2 Other driver gene and drug‐resistant mutations

In addition to *EGFR* mutations, other driver genes and potential drug‐resistant mutations were also revealed in the genomic profile, including *MET*, *ALK*, *ERBB2*, *KRAS*, ROS proto‐oncogene 1 (*ROS1*), *BRAF*, ret proto‐oncogene (*RET*), and neurotrophic receptor tyrosine kinase 1 (*NTRK1*). Overall, 155 mutations of at least one of the above genes were detected in 201 patients from 11 studies (Table S3). The most frequent mutation detected in CSF was *MET* amplification (37/155). *ALK* was the following mutated gene and was found in 33 patients, but this result needs to be interpreted cautiously because all the enrolled patients in the study of Zheng et al were *ALK*‐mutated.^14^ The followed genes with genomic alternations were *NTRK1* (23/155), *ERBB2* (18/155), and *BRAF* (14/155). *ERBB2* amplification was found in six patients. *NTRK* fusions and *BRAF* V600E are the rare driver mutations of NSCLC, while in the sequencing results of CSF, nearly all types of *NTRK1* and *BRAF* mutations were copy number amplifications (22/23, 13/14).

Tumor protein p53 (*TP53*) frequently occurred in CSF, with a detection rate of around 50%.^8^, ^9^, ^14^, ^16^, ^20^ Two studies reported a higher rate of *TP53* loss of heterozygosity (LOH) in CSF than in plasma (41.7% vs 13.9%, *p* < 0.001; 73.1% vs 7.7%, *P* < 0.001).^8^, ^9^ In those patients with *TP53* LOH or mutations, higher frequencies of genomic alternations in CNVs, *EGFR*, *MET*, and RB transcriptional corepressor 1 (*RB1*) were discovered. This profile was unique in CSF and might be related to resistance and the emergence of LM.

#### 3.3.3 Copy number variations

**FIGURE 3:**
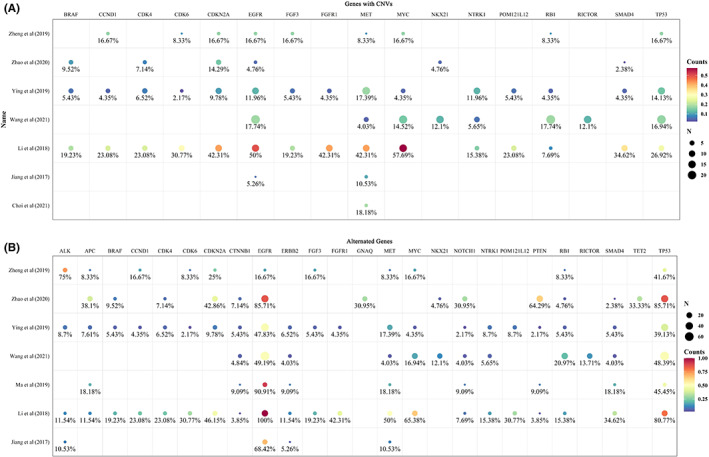
The frequency of copy number variations (CNVs) and alternations of genes reported in the included studies. The size of dots represents the number of patients with genomic alternations, and the color of dots represents the number of patients with the genomic alternations divided by the total number of patients in each study. (A) The 17 genes with CNVs that existed in more than 10 patients and their variated frequency. (B) The 25 genes with genomic alternations which existed in more than 10 patients and their alternated frequency.

Even though detecting CNVs is more challenging than SNVs due to technology issues,^28^ a significant number of CNVs were demonstrated and the majority of them were unique in CSF compared with plasma.^8^, ^9^, ^14^, ^16^ Seven studies reported CNVs of 64 genes, and 52 of them alternated in two or more patients. The most frequent gene was *EGFR*, followed by *TP53*. Other 15 genes had CNVs in more than 10 patients, which were MYC proto‐oncogene (*MYC)*, *MET*, *RB1*, cyclin‐dependent kinase inhibitor 2A (*CDKN2A*), *NTRK1*, NK2 homeobox 1 (*NKX2‐1*), cyclin‐dependent kinase 4 (*CDK4*), fibroblast growth factor receptor 1 (*FGFR1*), RPTOR independent companion of MTOR complex 2 (*RICTOR*), *BRAF*, SMAD family member 4 (*SMAD4*), cyclin D1 (*CCND1*), fibroblast growth factor 3 (*FGF3*), cyclin‐dependent kinase 6 (*CDK6*), and POM121 transmembrane nucleoporin like 12 (*POM121L12*) (Figure 3A). Most of the genes with CNVs also presented with other types of genomic alternations, while the fibroblast growth factor family genes such as *FGF3*, fibroblast growth factor 4 (*FGF4*), and fibroblast growth factor 19 (*FGF19*), and the cyclin family related genes such as *CCND1*, *CDK4*, and *CDK6* showed only copy number amplifications. Since somatic CNVs affect a larger fraction of the cancer genome than any other type of alterations does, the profile of CNVs in CSF of LM patients warrants further study.

#### 3.3.4 Alternated genes from sequencing of gene panels

In order to dig more information on genomic alternations in LM patients, we summarized the sequencing results from the studies which performed sequencing in gene panels instead of sequencing in several predefined genes. In summary, seven studies reported 120 genes with variations. Except for *EGFR*, the most frequently mutated gene was *TP53*. Ninety‐four genes mutated in two or more patients and 25 genes mutated in more than 10 patients (Figure 3B). Compared with the TCGA database, we found that 15 of these genes also appeared in the 25 most frequently mutated genes from the pan‐lung cancer data, while 10 genes were not among them, which were *MYC*, *NTRK1*, *NKX2‐1*, *RICTOR*, *CDK4*, *FGFR1*, *BRAF*, *CCND1*, *FGF3*, and *CDK6*, indicating an increased variation frequency of these genes in LM. Compared the 94 genes mutated in two or more patients to the list of CGC, we found that 10 genes did not belong to CGC but were detected in two or more studies, which were *POM121L12*, *FGF3*, *FGF19*, *FGF4*, neuron navigator 3 (*NAV3*), olfactory receptor family 4 subfamily A member 15 (*OR4A15*), casein kinase 2 alpha 1 (*CSNK2A1*), olfactory receptor family six subfamily F member 1 (*OR6F1*), BMP/retinoic acid inducible neural specific 3 (*BRINP3*), and thrombospondin type 1 domain containing 7A (*THSD7A*). These genes may promote oncogenic transformation and indicate a role in LM but need extensive available evidence.

### 3.4 Dynamic monitoring to guide treatment

Liquid biopsy is considered a dynamic method to trace genomic alternations and guide targeted treatment. Here, we summarized 14 representative cases (Table 3) and most of them achieved a good response after targeted treatment according to the detected mutations in CSF, especially those who switched treatment to third‐generation TKI after the emergence of T790M. The number and AFs of mutations in CSF tend to decrease after treatment, while the plasma sequencing usually shows the emergence of another mutation.

**TABLE 3:** The representative cases

Case	Study	Treatment before CSF sequencing	Mutations in CSF	Mutations in paired plasma	Treatment after CSF sequencing	Response						
						Improved condition	Improved MRI signs	Improved cytology	CSF sequencing	Decreased AFs in CSF	Plasma sequencing	Others
1	Frankel et al. (2018)	NA	Negative	NA	Chemotherapy, bevacizumab	NA	NA	NA	NA	NA	NA	PD after two cycles
2	Frankel et al. (2018)	NA	*EGFR* 19del	NA	Erlotinib	NA	NA	NA	*EGFR* T790M; *EGFR* 19del	NA	*EGFR* 19del	Started osimertinib
3	Li et al. (2018)	Gefitinib	*EGFR* T790M, *EGFR* 19del	*EGFR* T790M, *EGFR* 19del	Osimertinib	Yes	Yes	Yes	Negative T790M	19del: 39.02%–32.6%	NA	NA
4	Zheng et al. (2019)	Crizotinib	*EML4‐ALK*, *FSHR‐ALK*, *EGFR* CNG	Negative mutations	Brigatinib	Yes	NA	Yes	Only *EML4‐ALK*	*EML4‐ALK*: 91.88%–16.78%	NA	Relapsed after 16 months
5	Huang et al. (2019)	NA	*EGFR* 19del	*EGFR* 19del	Icotinib	No	2 months: Yes 4 months: No	NA	*EGFR* 19del	0.4%–0.03%	*EGFR* T790M	Started osimertinib; stable disease
6	Huang et al. (2019)	NA	*EGFR* L858R	Negative mutations	Erlotinib	NA	NA	NA	*EGFR* 19del	48%–67%	*EGFR* T790M	Started osimertinib; stable disease
7	Huang et al. (2019)	NA	*EGFR* L858R	Negative mutations	Icotinib	NA	NA	NA	NA	8.5%–1.6%		Stable disease
8	Huang et al. (2019)	Icotinib	*EGFR* T790M, *EGFR* L858R	*EGFR* T790M, *EGFR* L858R	Osimertinib	NA	NA	NA	*EGFR* T790M, *EGFR* L858R	T790M: 7%–0%, L858R: 75%–70%	*EGFR* T790M, *EGFR* L858R	Stable disease
9	Zheng et al. (2019)	Alectinib	*ALK* C1156F, *ALK* G1202R	Negative mutations	Brigatinib	NA	NA	NA	NA	NA	NA	Relapsed after 1 month
10	Ma et al. (2020)	NA	*EGFR* G719A	NA	Afatinib	NA	Yes	NA	NA	55.6%–23.1%	NA	CEA: 9470–1590 ng/ml
11	Ma et al. (2020)	NA	*EGFR* L861Q	NA	Afatinib	NA	Yes	NA	NA	NA	NA	CEA: 786.9–98.1 ng/ml
12	Ma et al. (2020)	NA	*EGFR* L861Q	NA	Afatinib	NA	NA	NA	NA	NA	NA	CEA: 168.3–35.4 ng/ml
13	Zhao et al. (2020)	Icotinib	*EGFR* T790M, *EGFR* 19del	NA	Osimertinib	Yes	Yes	Yes	Negative mutations	NA	NA	Nearly 3 years survival
14	Choi et al. (2020)	Olmutinib	*EGFR* C797S	*EGFR* 19del	Erlotinib	Yes	Yes	NA	*EGFR* 19del, *EGFR* C797S	19del: dropped	*EGFR* 19del, *EGFR* C797S	Increased T790M in CSF and plasma 17.8 weeks later; started osimertinib combined with erlotinib

Abbreviations: AF, allele fraction; CNG, copy number gain; CSF, cerebrospinal fluid; EGFR, epidermal growth factor receptor; MRI, magnetic resonance imaging; NA, not available.

In the studies, a higher detection rate of T790M was found in extracranial lesions and plasma than in CSF.^8^, ^9^, ^12^, ^23^ After changing gefitinib to erlotinib, which has a higher concentration in CSF, more T790M was detected after intracranial disease progression,^18^ indicating the association between the occurrence of T790M and the exposure level of TKI in CSF. When using the first‐ or second‐generation TKI, the level of classical *EGFR* mutations in CSF dropped, while after that, the subsequent change was the increase of T790M level in plasma, which occurred earlier than that in CSF.^21^, ^24^ The same phenomenon was also found in patients with C797S mutation in CSF who received first‐ or second‐generation TKI.^24^ However, after third‐generation TKI treatment failure, the mutated rate of T790M in CSF became higher than in plasma,^23^ which illustrated the third‐generation TKI has a certain degree of CNS permeability, but the response of LM is not as obvious as extracranial lesions. Due to the different CNS permeability of TKI, dynamic changes of T790M in plasma seem to be more sensitive than that in CSF, even though there was a definite progression of LM. Therefore, sequencing of plasma needs to be combined with CSF to guide treatments for LM patients.

## 4 DISCUSSION

In this review, we summarize the existing literature on the topic of sequencing of CSF in NSCLC patients with LM. We found sequencing of CSF performed well and revealed a unique genomic profile. We also summarized the cases that used CSF sequencing to guide treatments and suggested that it was better to combine the sequencing of plasma together.

Plasma and CSF have been widely compared as samples for liquid biopsy in LM patients. Here, we found CSF performed better than plasma. The phenomenon attributes to some possible reasons. First, more interference of background gene fragments from normal blood cells existed in plasma than in CSF. Second, LM tumor cells are bathed by CSF, so the DNA fragments are released into CSF directly. However, the cells and cfDNA in CSF need to be absorbed through the arachnoid villi into the superior sagittal sinus every 4 h and then get into the systemic circulation. Third, the interference from extracranial lesions could be prevented by the blood–brain barrier and blood–CSF barrier.^6^, ^29^ There are several sequencing samples in CSF, including cell precipitates, CTC, and cfDNA. Studies suggested the concordance of different samples in plasma was not high.^30^ However, the difference in CSF was rarely discussed and needs further study. The current limitation in sequencing cells in CSF corresponds to technical defects, associated with low abundance of CTC in biofluids, inevitable damage to tumor cells during sample preparation, and additional complex assessment of bioinformatics analyses.^31^, ^32^, ^33^ Therefore, if qualified cell samples in CSF are too scarce, cfDNA may be more suitable for patients with LM.

Tumor metastasis is an evolutionary process, and the acquired genomic alterations of metastatic carcinoma during disease evolution induce treatment resistance and accelerate dissemination,^34^, ^35^ although previous studies mainly focused on the brain parenchyma metastasis.^36^, ^37^ Here, genomic alterations of LM were detected in 120 genes and most of them existed in the list of CGC, possessing a definite activity in cancer development. However, 10 genes were not listed but were reported in over two studies and two patients, which may play special roles in the evolutionary process of LM. Of them, *OR4A15* and *OR6F1* belong to the olfactory receptor family, which can pair with the molecular secreted from neighboring neurons and is also highly expressed in different cancer tissues.^38^, ^39^, ^40^, ^41^ *NAV3* and *BRINP3* are predominantly expressed in the nervous system and involved in neuron proliferation and regeneration. It is important to explore whether these mutations are interference of background genes in CSF or an implication of connections between LM tumor cells and neurons.

A common characteristic across studies is the number of CNVs detected in CSF, although the large fragment alternations were difficult to recognize during sequencing. We speculated that indicated the potential existence of genomic instability and might be the underlying cause of the difference in LM. *TP53* LOH uniquely occurred in CSF rather than plasma, accompanied by more genomic mutations and CNVs. Aneuploidy, another trigger of genomic instability, is also widely detected in patients in CSF from LM patients.^42^ The coexistence of genomic instability and aneuploidy could promote each other and lock in a vicious cycle, leading to genomic copy number changes. The CNVs facilitate tumor cells to acquire phenotypes to survive under selective pressure such as anti‐cancer therapy.^43^ Considering the special environment in CSF, we suspected the continuous change of genome may also endow LM tumors with the ability to adapt to the sparse micronutrients and hypoxia circumstances. In our review, CNVs were reported in 64 genes and the most frequent CNV was *MET* amplification, which was found associated with treatment resistance and brain metastasis in NSCLC. Most genes with CNVs also mutated, emphasizing the importance of genomic alterations in these genes and their relevant pathways. While the genes belonging to the fibroblast growth factor family and cyclin family showed a specific amplification, which indicated an enhanced function in the cell cycle and survival activities in LM.

In the representative cases, most patients improved in both brain imaging and CSF cytology after receiving TKI. Dynamic monitoring revealed mutation subtypes and AFs of *EGFR* changes in CSF along with treatments. Besides, mutations in CSF might provide clues to different treatment responses, such as T790M in TKI‐naive patients and bypass activations including *MET* amplification and *ERBB2* amplification.^16^ It is obvious that the changes of *EGFR* subtypes in CSF and plasma were not simultaneous and T790M occurred earlier in plasma than in CSF after first‐ or second‐generation TKI therapy. The phenomenon may attribute to the followings: (1) the intracranial lesions were not exposed to the concentration of first‐ or second‐generation TKI that was enough to kill the cancer cells due to the obstruction of the blood–CSF barrier and (2) the blood–brain barrier and blood–CSF barrier also obstruct T790M mutations into CSF from systemic circulation. Furthermore, the difference in genomic alternations between CSF and plasma can be affected by clinical features, radiotherapy history, or TKI treatment of patients,^25^ demonstrating the importance of sequencing in both plasma and CSF for treatment decisions.

In conclusion, like tissue biopsy, sequencing of CSF in LM patients can guide targeted treatments and reveal resistance mutations and also provides a dynamic method to monitor intracranial tumor response. Furthermore, it reveals a unique genomic profile including mutations in driver genes and drug‐resistant genes, *TP53* LOH, a number of CNVs, and more mutated genes than plasma, indicating the potential existence of genomic instability in LM tumors. The sequencing of CSF in LM patients seems to provide more comprehensive information than we expected and the biological significance behind the genomic alternations needs further study.

## AUTHOR CONTRIBUTIONS

Tianqi Gao: Have made substantial contributions to conception and design, acquisition of data, or analysis and interpretation of data; been involved in drafting the manuscript or revising it critically for important intellectual content; agreed to be accountable for all aspects of the work in ensuring that questions related to the accuracy or integrity of any part of the work are appropriately investigated and resolved. Fengxi Chen: have made substantial contributions to conception and design, acquisition of data, or analysis and interpretation of data; been involved in drafting the manuscript or revising it critically for important intellectual content. Man Li: have made substantial contributions to conception and design, acquisition of data, or analysis and interpretation of data; Given final approval of the version to be published. Each author should have participated sufficiently in the work to take public responsibility for appropriate portions of the content; Agreed to be accountable for all aspects of the work in ensuring that questions related to the accuracy or integrity of any part of the work are appropriately investigated and resolved.

## CONFLICT OF INTEREST

No potential conflict of interest relevant to this article was reported.

## ETHICS STATEMENT

Our study is secondary research, and all data presented were from published studies. So, there is no need for ethical approval.

## Supporting information


Appendix S1

Table S1

## Data Availability

The data that support the findings of this study are available from the corresponding author upon reasonable request.
