# Supplementary material for: Sequencing of cerebrospinal fluid in non‐small‐cell lung cancer patients with leptomeningeal metastasis: A systematic review
Source: Cancer Med. 2022 Aug 24;12(3):2248–61. doi: 10.1002/cam4.5163 (PMC9939157; doi:10.1002/cam4.5163)
Supplement: Supplementary file 1 — Appendix S1 [file CAM4-12-2248-s001.docx]

Supplementary File.

Search strategies in the PubMed database

| **Search Criteria** | **Search Algorithm** | **Yield**  **(December 31, 2021)** |
| --- | --- | --- |
| 1 | ‘cancer’ OR ‘neoplasm’ OR ‘carcinoma’ OR ‘tumour’ OR ‘tumor’ | 4,749,686 |
| 2 | ‘metastasis’ OR ‘metastatic’ OR ‘metastasize’ OR ‘metastasis’ OR ‘metastases’ | 1,351,378 |
| 3 | ‘leptomeningeal’ OR ‘leptomeninges’ OR ‘pia mater’ OR ‘meningeal’ OR ‘meninges’ OR ‘carcinomatous meningitis’ OR ‘neoplastic meningitis’ | 129,123 |
| 4 | ‘cell-free fluid sample’ OR ‘liquid biopsy’ OR ‘circulating tumor cell’ OR ‘circulating tumour cell’ OR ‘CTC’ OR ‘cell-free tumor DNA’ OR ‘ctDNA’ | 52,280 |
| 5 | #1 AND #2 AND #3 AND #4 | 128 |

Search strategies in the Medline database

| **Search Criteria** | **Search Algorithm** | **Yield**  **(December 31, 2021)** |
| --- | --- | --- |
| 1 | TS=(NSCLC OR non small cell lung cancer OR lung cancer) | 256,918 |
| 2 | TS=(leptomeningeal OR leptomeninges OR pia mater OR meningeal OR meninges OR carcinomatous meningitis OR neoplastic meningitis) | 48,249 |
| 3 | TS=(cell-free fluid sample OR circulating tumor cell OR ctDNA OR cell-free tumor DNA OR circulating tumour cell OR CTC) | 40,892 |
| 4 | #1 AND #2 AND #3 | 49 |

Search strategies the European Society for Medical Oncology

| **Search Criteria** | **Filter by** | **Yield**  **(December 31, 2021)** |
| --- | --- | --- |
| 1 | Leptomeningeal metastases | 33 |
| 2 | Liquid biopay | 31 |
| 3 | ctDNA | 17 |
| 4 | CSF | 1 |
| 5 | Screened total retrieved articles | No eligible articles |

Search strategies the European Society for Medical Oncology

| **Search Criteria** | **Filter by** | **Yield**  **(December 31, 2021)** |
| --- | --- | --- |
| 1 | Topic=cancers> lung cancer> non small cell lung carcinoma AND metastatic disease> leptomeningeal metastases | |
| 2 | Type=Abstracts & Presentations AND Journals | |
| 3 | Leptomeningeal metastases | 54 |
| 4 | Liquid biopsy | 67 |
| 5 | ctDNA | 36 |
| 6 | Screened total retrieved articles | No eligible articles |
